# Long non-coding RNA TUG1 promotes cell progression in hepatocellular carcinoma via regulating miR-216b-5p/DLX2 axis

**DOI:** 10.1186/s12935-019-1093-6

**Published:** 2020-01-07

**Authors:** Qun Dai, Jingyi Deng, Jinrong Zhou, Zhuhong Wang, Xiao-feng Yuan, Shunwen Pan, Hong-bin Zhang

**Affiliations:** 1 Department of Pediatric, The Third Affiliated Hospital of Sun Yat-Sen University, Guangzhou, 510630 China; 2 Department of Emergency, The Third Affiliated Hospital of Sun Yat-Sen University, Guangzhou, 510630 China; 3 Department of General Intensive Care Unit Medicine, The Third Affiliated Hospital of Sun Yat-Sen University, Guangzhou, 510630 China; 4 Department of Laboratory Medicine, The Third Affiliated Hospital of Sun Yat-Sen University, No. 600, Tianhe Road, Guangzhou, 510630 China

**Keywords:** HCC, TUG1, miR-216b-5p, DLX2

## Abstract

**Background:**

Accumulating evidence indicates that the long noncoding RNA taurine upregulated gene 1(TUG1) plays a critical role in cancer progression and metastasis. However, the overall biological role and clinical significance of TUG1 in hepatocellular carcinoma (HCC) remain largely unknown.

**Methods:**

The expressions of TUG1, microRNA-216b-5p and distal-less homeobox 2 (DLX2) were detected by Quantitative real-time polymerase chain reaction (qRT-PCR). The target relationships were predicted by StarBase v.2.0 or TargetScan and confirmed by dual-luciferase reporter assay. The cell growth, apoptosis, migration and invasion were detected by 3-(4,5-Dimethylthiazol-2-yl)-2,5-diphenyltetrazolium bromide (MTT), Flow cytometry and Transwell assays, respectively. All protein expression levels were detected by western blot. Tumor xenografts were implemented to explore the role of TUG1 in vivo.

**Results:**

We found that there was a marked rise in TUG1 expression in HCC tissues and cells, and knockdown of TUG1 repressed the growth and metastasis and promoted apoptosis of HCC cells. In particular, TUG1 could act as a ceRNA, effectively becoming a sink for miR-216b-5p to fortify the expression of DLX2. Additionally, repression of TUG1 impared the progression of HCC cells by inhibiting DLX2 expression via sponging miR-216b-5p in vitro. More importantly, TUG1 knockdown inhibited HCC tumor growth in vivo through upregulating miR-216b-5p via inactivation of the DLX2.

**Conclusion:**

TUG1 interacting with miR-216b-5p contributed to proliferation, metastasis, tumorigenesis and retarded apoptosis by activation of DLX2 in HCC.

## Highlights

- TUG1 was validated to act as a molecular sponge of miR-216b-5p.
- The targeted relationship between DLX2 and miR-216b-5p was first verified.
- TUG1 promoted HCC cell progression via miR-216b-5p/DLX2 axis.

## Background

Hepatocellular carcinoma (HCC) is a widespread health-damaging neoplasm and ranks the third major reason which gives rise to cancer-associated deaths all over the world [1]. Although many recent studies in big data genomics and molecular biology have revealed a lot of carcinogenic factors and molecular regulatory mechanisms of HCC, most patients have been diagnosed at an advanced stage, and the tumor has metastasized extensively and is prone to recurrence after surgery [2, 3]. It has been reported that HCC accounts for more than 90% of primary liver cancers [4]. Therefore, it is necessary to reveal the relevant marker molecules and cellular mechanisms of HCC.

Non-coding RNAs (ncRNAs), containing widely studied microRNAs (miRNAs) and latterly identified long non-coding RNAs (lncRNAs), have been served as oncogenes or tumor suppressor genes for various tumors, becoming new potential therapeutic targets [5, 6]. LncRNAs are known as RNA molecules longer than 200 nucleotides without coding capacity [7]. MicroRNAs, which also have no coding ability, are small RNAs of about 22 nucleotides in length [8], have been shown to regulate the development of multiple cancers [9]. lncRNAs and miRNAs have functional roles in numerous cellular and molecular biological processes. It’s worth mentioning that lncRNAs could play a part of competitive endogenous RNAs (ceRNAs) to interplay with miRNAs and regulate the miRNA targeted gene expression [10].

Latterly, taurine upregulated gene 1 (TUG1) expression was proved to be enormously elevated in most cancers, for instance prostate cancer [11], breast cancer [12], endometrial cancer [13] and cervical cancer [14]. More than that, TUG1 was aberrantly aggrandized in HCC tissues, and it could promote cell growth and tumor formation by targeting KLF2 [15]. For all this, the specific molecular regulation mechanism of TUG1 in HCC progression remains unclear. It’s worth noting that lncRNAs could impede miRNA molecular functions by complementarity of sequences, consequently removing the inhibitory influence of miRNA with target genes [16]. It is reported that miR-216b-5p was exceptionally constrained in prostate cancer and coulg be inhibited by lncRNA Linc00518 [17]. Based on these date, we speculated whether TUG1 could serve as a ceRNA of miR-216b-5p in HCC. In addition, a recent study showed that high expression of DLX2 was related to terminal stages of cancer and needy prognosis in patients with HCC [18]. Interestingly, DLX2 might be a target gene for miR-216b-5p was predicted by TargetScan. Hence, we have made a bold hypothesis that TUG1 might participate in the regulation of HCC progression through the miR-216b-5p/DLX2 axis.

In this research, we mainly aimed to investigate the carcinogenesis mechanism of TUG1 in HCC, providing a new diagnostic marker gene and therapeutic target for HCC.

## Materials and methods

### Tissue samples and cell culture

Fresh HCC tissues and adjacent normal tissues were harvested from 40 patients at The Third Affiliated Hospital of Sun Yat-Sen University. Patients had not received any treatment before the surgery and wrote an informed consent. Tissues were separated and kept in liquid nitrogen. The research was approved by the Ethics Committee of The Third Affiliated Hospital of Sun Yat-Sen University.

Human HCC cell lines (HCC-2, Hep3B, Huh7 and CLC1) were obtained from Beinuo Biotech (Shanghai, China) and normal cell line (THLE-2) was bought from American Type Culture Collection (ATCC; Manassas, VA, USA). Cells were grown in DMEM (Thermo Fisher Scientific, Waltham, MA, USA) including 10% fetal bovine serum (Thermo Fisher Scientific), and 1% penicillin/streptomycin (Invitrogen, Carlsbad, CA, USA) at 37 °C in 5% CO_2_.

### Transfection assay

MiR-216b-5p mimics and miR-NC, miR-216b-5p inhibitor and anti-miR-NC, TUG1 siRNA and si-NC, sh-TUG1 and sh-NC were acquired from RiboBio (Guangzhou, China). Overexpression vectors (TUG1 and DLX2) and control (pcDNA) were bought from SyngenTech (Beijing, China). The Lipofectamine 3000 (Invitrogen) was applied to transfection.

### Reverse transcription-quantitative polymerase chain reaction (RT-qPCR)

Total RNA was separated through the Trizol reagent (Invitrogen). SYBR Green Real-time PCR Master Mix (Takara, Dalian, China) was applied to reverse transcribe RNA into cDNA, followed by the ABIPRISM 7900 Sequence Detection System (Applied Biosystems, Foster City, CA, USA). TUG1 and DLX2 expression were standardized to glyceraldehyde-3-phosphate dehydrogenase (GAPDH). MiR-216b-5p expression was standardized to U6. The results were computed by 2^−∆∆Ct^ method. Respectively, the following primers were applied: TUG1 (ID: 55000) (Forward: 5′-CTGAAGAAAGGCAACATC-3′; Reverse: 5′- GTAGGCTACT ACAGGATTTG-3′); DLX2 (ID: 1746) (Forward: 5′-CTCTGCCTGCCTCATAAGG-3′; Reverse: 5′-ATCGTAAGAACAGCGCAACC-3′); GAPDH (Forward: 5′-GGAGCGAGATCC CTCCAAAAT-3′; Reverse: 5′-GGCTGTTGTCATACTTCTCATGG-3′); miR-216b-5p (ID: 100126319) (Forward: 5′-GAAATCTCTGCAGGCAAATGTG-3′; Reverse: 5′-GTGCAGGGTCCGAGGT-3′); U6 (Forward: 5′-CTCGCTTCGGCAGCACA-3′; Reverse: 5′-AACGCTTCACGAATTTGCGT-3′).

### Dual luciferase reporter assay

TUG1 wild type (TUG1-WT) or mutant type (TUG1-MUT) containing miR-216b-5p interacting site or not were transfected into cells with miR-216b-5p mimics or miR-NC by using Lipofectamine 2000 (Invitrogen). DLX2-3′UTR-WT or DLX2-3′UTR-MUT were also co-transfected into cells with miR-216b-5p mimics or miR-NC. After transfection for 36 h, the luciferase activity was determined using the Dual-luciferase reporter system (Promega, Madison, WI, USA).

### Cell proliferation assay

3-(4,5-Dimethylthiazol-2-yl)-2,5-diphenyltetrazolium bromide (MTT) (KyeGEN BioTECH, Nanjing, China) was used to detect cell proliferation as described previously [19]. Cells were inoculated with the density of 1 × 10^5^ cells/well in 96-well plate (Costar, Corning, NY, USA). At designated times (0 h, 24 h, 48 h, 72 h, 96 h, 120 h) transfection, Next, the MTT (5 mg/mL) of 10 µL was added to each well and cells were incubated for 4 h. Discarding supernatant later, the dimethyl sulfoxide (DMSO) of 150 µL was also added to solubilize the formazan salt. Finally, the absorbance at 570 nm was detected by using an Elx800 Reader (BioTek Instruments Inc., Winooski, VT, USA).

#### Cell cycle and apoptosis analysis

Cell cycle and apoptosis assays were carried out as described previously [20]. The transfected cells were collected and washed twice at 4 °C with PBS, followed by fixation using cold 70% ethanol at 4 °C overnight. After removed ethanol, cells were washed and resuspended in propidium iodide (PI) solution containing 50 μg/mL RNase and 100 μg/mL PI (Keygen, Nanjing, China) at 37 °C for 20 min without night. Cells were washed and analyzed using flow cytometry to detect the DNA content of the stained cells. For cell apoptosis detection. Cells were stained with Annexin V-FITC (Keygen) and PI for 15 min at room temperature. Finally, Flow cytometry was performed to determine the percentage of apoptotic cells.

### Western blot assay

Western blot assay was performed as described previously [21]. Tissues and cell lysates were prepared by using RIPA Reagent (Beyotime, Shanghai, China) and the concentration was measured by BCA kit (Thermo Fisher Scientific). Proteins were loaded on 10% SDS-PAGE and were transferred onto PVDF membranes (Thermo Fisher Scientific). The membranes were immersed in 5% skim milk powder for 2 h, and then incubated at 4 °C overnight with anti-CyclinD1 (1:200, Abcam, Cambridge, MA, USA), anti-CDK4 (1:2000, Abcam), anti-PARP (1:1000, Cell Signaling Technology, Shanghai, China), anti-Cleaved PARP (1:1000, Abcam), anti-Cleaved caspase-3 (1:500, Abcam), anti-E-cadherin (1:1000, Cell Signaling Technology), anti-N-cadherin (1 µg/mL, Abcam), anti-Vimentin (1:1000, Abcam), anti-DLX2 (1:500, Abcam), Ki67 (1:1000, Abcam) or anti-GAPDH (1:2500, Abcam). Next, membranes were washed and probed with the HRP-labeled secondary antibody (Abcam) for 1 h at 37 °C. Membranes were visualized via the ECL regent (Amersham Biosciences, Buckinghamshire, UK).

### Cell migration and invasion assay

Migration and invasion were detected by transwell assay with or without Matrigel (Corning Life Sciences, Corning, NY, USA) as described previously [22]. Cells were fixed with 100 mL of serum-free medium and seeded into the upper chamber, the DMEM of 600 mL containing 10% FBS was added to the lower chamber. After incubation for 24 h, cells were attached to the lower surface of the upper chamber were treated with ethanol and dyed with 0.1% crystal violet and counted under a microscope.

### Tumor xenograft assay

Primary tumors were obtained by injecting 6-week-old nude female mice subcutaneously with 4 × 10^6^ Huh7 cells transfected with sh-TUG1 (n = 4 per group) or sh-NC. One week later, tumor growth was examined every 5 days for 5 weeks. Then, mice were sacrificed and tumors were weighted. The RNA and protein from tumor tissues were extracted for RT-qPCR and western blot analysis. The animal experimental procedures were conducted with the permission of the Animal Care and Use Committee of The Third Affiliated Hospital of Sun Yat-Sen University.

### Statistical analysis

Statistics were analyzed with SPSS 22.0 software. The correlation between the two groups was analyzed by Pearson’s coefficient. All results were presented as mean ± standard deviation (SD) and performed at least three self-governed experiments. The criterion for statistical significance was taken as *P < 0.05.

## Results

### TUG1 was up-regulated and miR-216b-5p was down-regulated in HCC tissues and cell lines

**Fig. 1:**
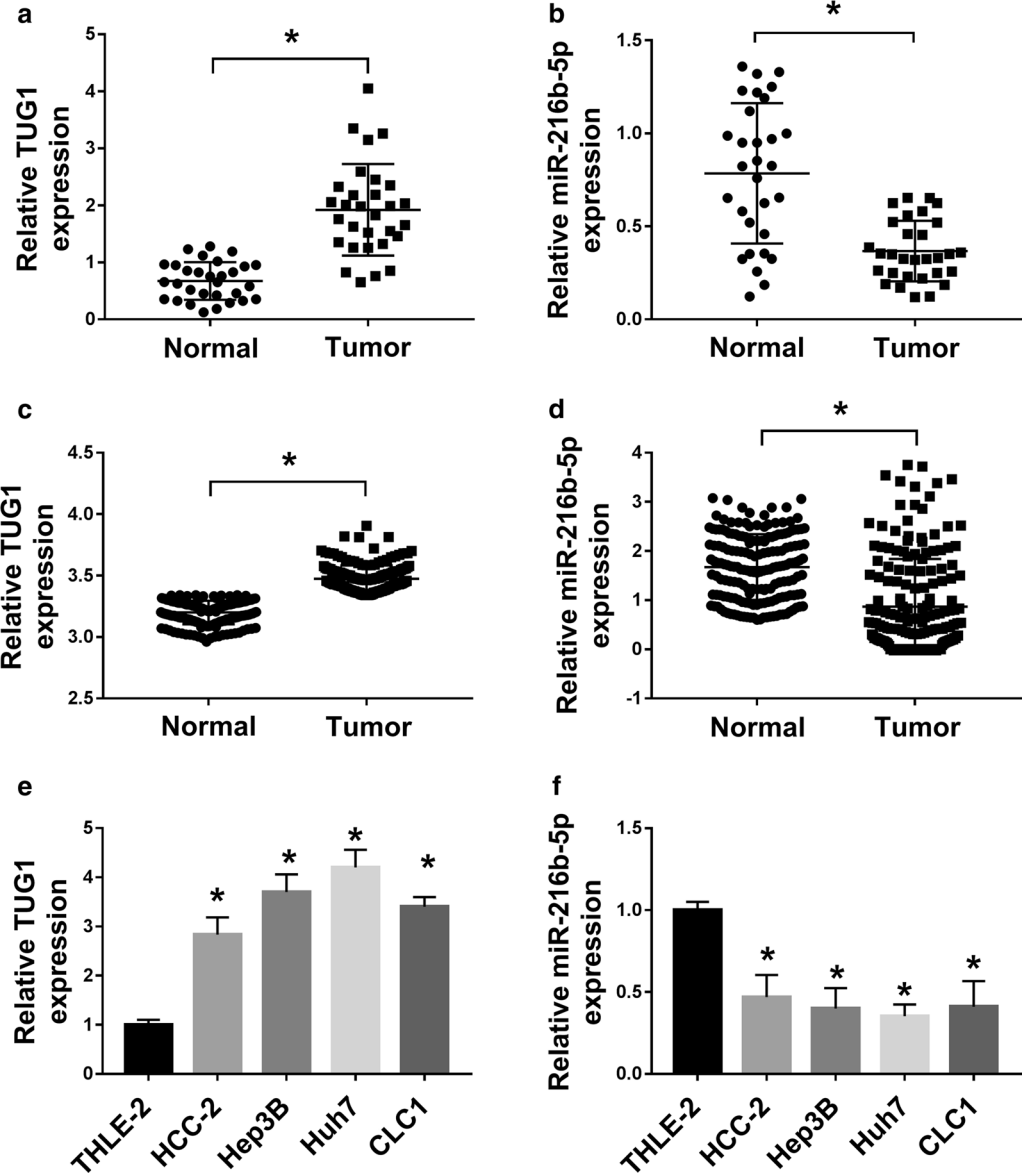
TUG1 expression was up-regulated and miR-216b-5p was down-regulated in HCC tissues and cells. **a**, **b** The expression levels of TUG1 and miR-216b-5p in 40 pairs of HCC tissues and matched noncancerous tissues were measured by qRT-PCR. **c**, **d** The expression levels of TUG1 and miR-216b-5p were analyzed in TCGA datasets of HCC patients. **e**, **f** The expression levels of TUG1 and miR-216b-5p in human normal hepatocyte cell line THLE-2 and HCC cell lines HAK-2,Hep3B,Huh7 and LC05 were detected using qRT-PCR. *P < 0.05

To explore the biological role of lncRNA TUG1 in HCC, TUG1 expression in 40 HCC tissues was measured by qRT-PCR. TUG1 expression was significantly upregulated in HCC tissues compared with that in normal tissues (Fig. 1a). Simultaneously, we found that the expression of miR-216b-5p was enormously decreased compared with normal tissues (Fig. 1b). Consistently, we observed an increase in TUG1 expression and a decrease in miR-216b-5p expression in HCC patients through TCGA datasets (Fig. 1c, d). We next performed qRT-PCR analysis to examine the expression levels of TUG1 and miR-216b-5p in various cell lines. The result showed that TUG1 expression was exceptionally facilitated in four human HCC cell lines HAK-2,Hep3B,Huh7 and LC05 compared with normal liver cell line THLE-2 (Fig. 1e), and that the miR-216b-5p expression was exceptionally constrained in four HCC cell lines than that in normal liver cell (Fig. 1f). These results implied that TUG1 was an oncogene and miR-216b-5p was a tumor suppressor gene, which might have the opposite effect in HCC. Additionally, the Hep3B and Huh7 cells were used for further experiments.

### TUG1 could act as a molecular sponge for miR-216b-5p

**Fig. 2:**
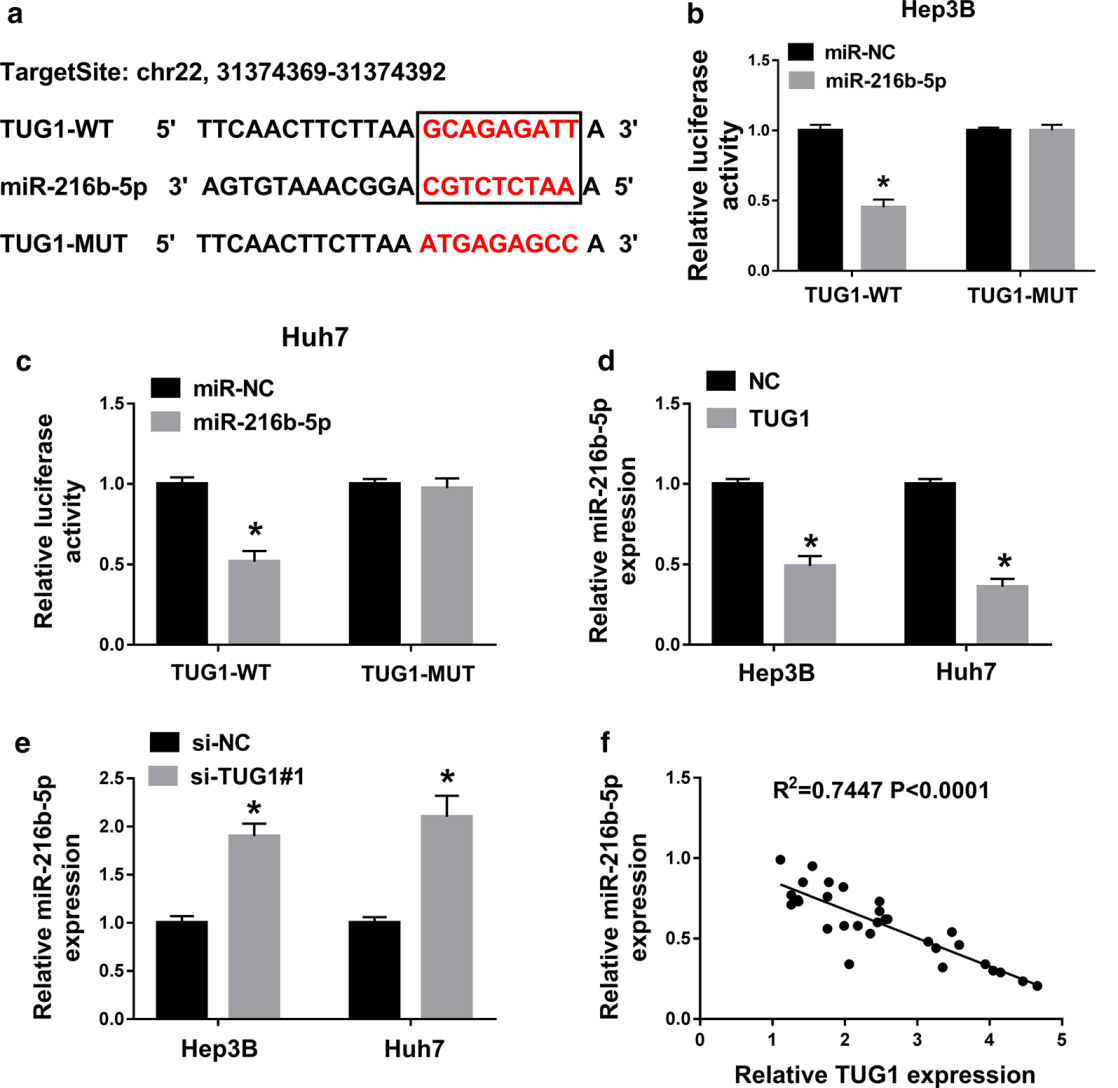
TUG1 acted as a molecular sponge for miR-216b-5p. **a** The binding sites of TUG1 and miR-216b-5p were predicted by starBase v2.0. **b**, **c** Relative luciferase activity of Hep3B and Huh7 cells co-transfected TUG1-WT or TUG1-MUT with miR-216b-5p or miR-NC was checked by dual-luciferase reporter assay. **d**, **e** MiR-216b-5p expression in Hep3B and Huh7 cells transfected with TUG1, NC, si-TUG1 or si-NC was measured by qRT-PCR. **f** Statistical correlations between the expression levels of TUG1 and miR-216b-5p were analyzed by Spearman’s test in SPSS 20.0 software (R^2^ = 0.7447, *P *< 0.0001). *P < 0.05

Considering that TUG1 and miR-216b-5p have opposite expression patterns in HCC, we suspected that there might be a targeted relationship between them. We found that TUG1 and miR-216b-5p have potential binding sites by StarBase v.2.0 (Fig. 2a). Then, the interaction between TUG1 and miR-216b-5p was confirmed by dual-luciferase assay. The results showed that the luciferase activity of TUG1-WT in Hep3B and Huh7 cells was decreased by the miR-216b-5p mimic (miR-216b-5p), but the TUG1-MUT activity was not significantly changed (Fig. 2b, c). To further study the relationship between TUG1 and miR-216b-5p, we overexpressed or interfered with TUG1 in Hep3B and Huh7 cells, and then detected the changes in the expression of miR-216b-5p. The results showed that miR-216b-5p was significantly decreased after overexpression of TUG1, and miR-216b-5p was notably augmented after cells transfected with si-TUG1 (Fig. 2d, e). More than that, a significant inverse correlation between TUG1 and miR-216b-5p was also observed (R2 = 0.7447, P < 0.0001) (Fig. 2f). These data demonstrated that TUG1 directly interacted with miR-216b-5p.

### Knockdown of TUG1 could repress the growth of HCC cells and promote apoptosis by regulation of miR-216b-5p

**Fig. 3:**
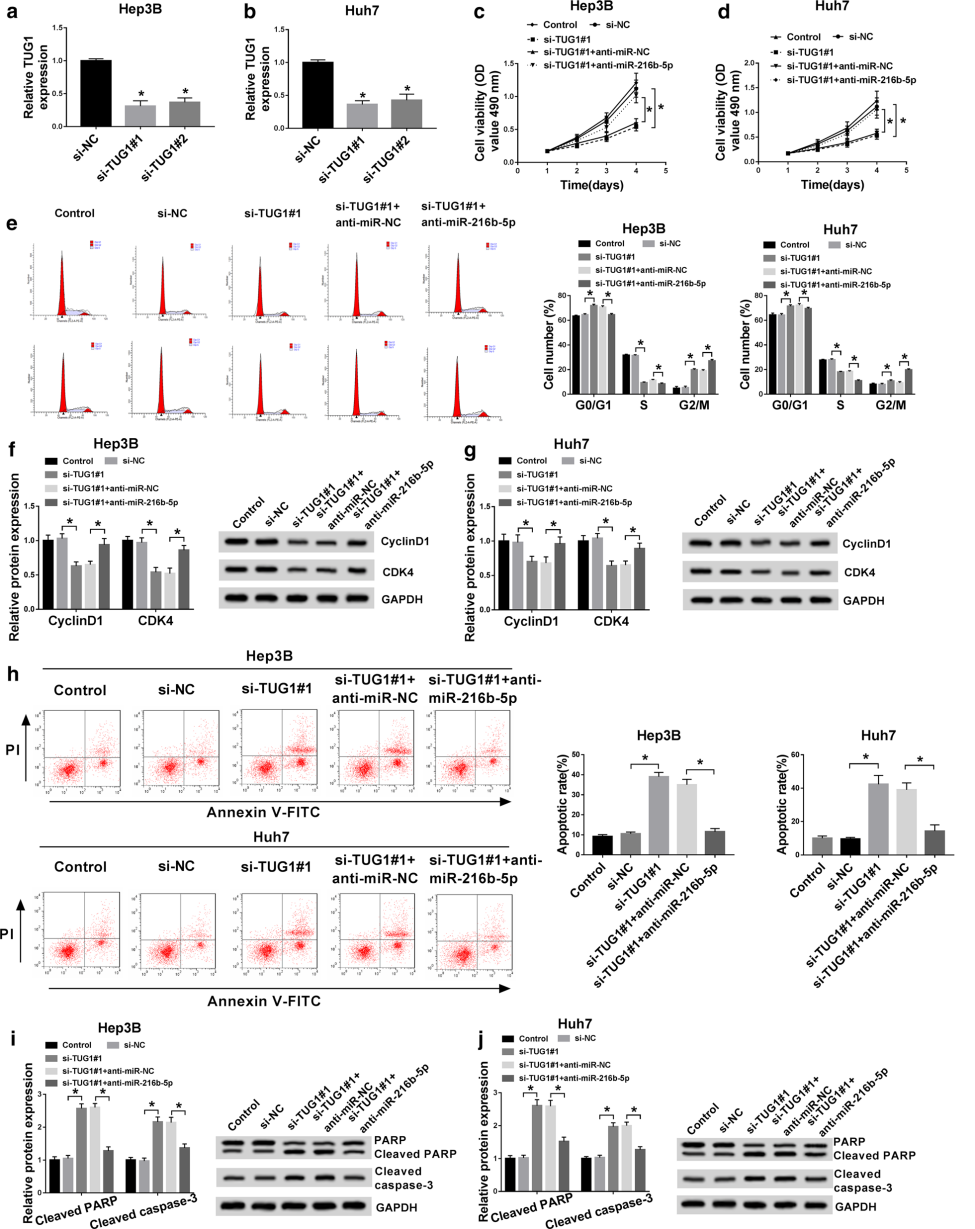
The effect of interference with TUG1 on HCC cells could be restored by anti-miR-216b-5p. **a**, **b** The TUG1 expression in Hep3B and Huh7 cells transfected with si-NC, si-TUG1#1 or si-TUG1#2 was tested by qRT-PCR. **c**–**e** The proliferation and cell cycle of Hep3B and Huh7 cells transfected with control, si-NC, si-TUG1#1, si-TUG1#1 + anti-miR-NC or si-TUG1#1 + anti-miR-216b-5p were detected by MTT and Flow cytometry. **f**, **g** The protein levels of CyclinD1 and CDK4 were checked by western blot. **h** The apoptosis of transfected Hep3B and Huh7 cells was determined by Flow cytometry. **f**, **g** Western blot assay was used to measure the levels of PARP, cleaved PARP and cleaved caspase-3. *P < 0.05

To determine whether TUG1 and miR-216b-5p expression levels were associated with HCC cell progression, we performed TUG1 loss-of-function experiments in Hep3B and Huh7 cells. Firstly, two siRNAs were synthesized and transfected into cells, and TUG1 level was tested for knockdown efficiency (Fig. 3a, b). One of the most efficient siRNAs, si-TUG1#1 was selected for the following experiments. MTT assay demonstrated that TUG1 silencing hindered both Hep3B and Huh7 cells proliferation, whereas the inhibitory effect of si-TUG1 on cell proliferation was rescued by the addition of anti-miR-216b-5p (miR-216b-5p inhibitor) (Fig. 3c, d). Cell cycle analysis showed that knockdown of TUG1 significantly increased the proportion of Hep3B and Huh7 cells in G0/G1 and G2/M phases, and decreased cell proportion in S phase, while repression of miR-216b-5p reversed these effects (Fig. 3e). We then examined the changes in expression of cell cycle marker proteins CyclinD1 and CDK4 by western blot. The data revealed that the inhibition effects of si-TUG1 on levels of CyclinD1 and CDK4 were attenuated by co-transfection of anti-miR-216b-5p (Fig. 3f, g). Furthermore, the number of apoptotic cells was significantly higher in si-TUG1-treated Hep3B and Huh7 cells compared with si-NC-treated cells by Flow cytometry, however, the promotion effect of si-TUG1 on cell apoptosis was also rescued by adding anti-miR-216b-5p (Fig. 3h). What’s more, we found that TUG1 depletion enhanced cleaved PARP and cleaved caspase-3 protein levels, whereas when miR-216b-5p was silenced, the levels of cleaved PARP and cleaved caspase-3 were reduced (Fig. 3i, j). Collectively, these data indicated that TUG1 facilitated cell growth and hampered apoptosis in HCC cells by targeting miR-216b-5p.

### TUG1 knockdown suppressed the metastasis of HCC cells by targeting miR-216b-5p

**Fig. 4:**
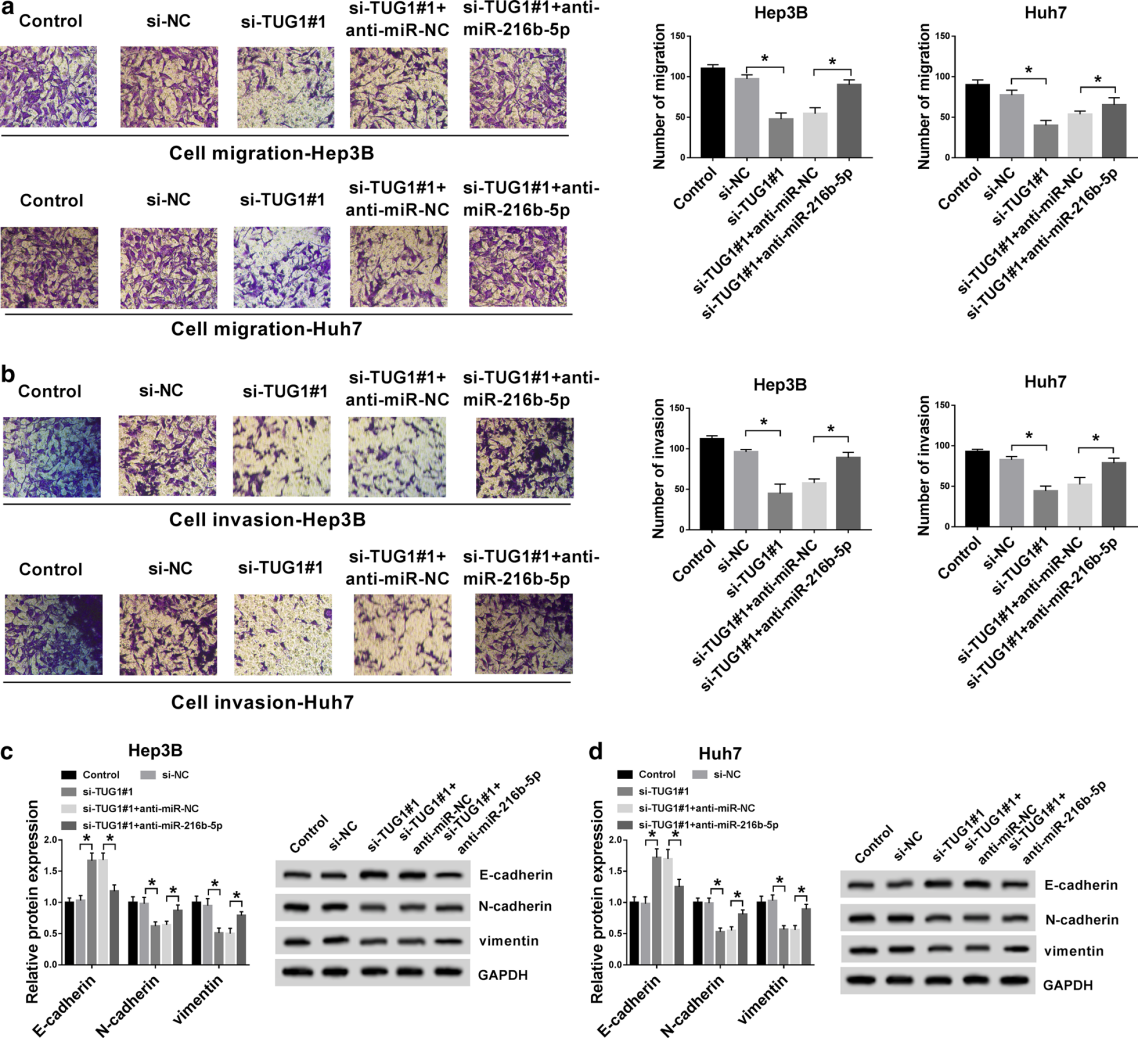
TUG1 knockdown suppressed the metastasis of HCC cells by targeting miR-216b-5p. **a**, **b** The migration and invasion of Hep3B and Huh7 cells were detected by Transwell assay. **c**, **d** The protein levels of E-cadherin, N-cadherin and Vimentin were examined by western blot. *P < 0.05

Subsequently, Transwell assay revealed that down-regulation of miR-216b-5p partially reversed the si-TUG1-induced inhibitory effects on migration and invasion of Hep3B and Huh7 cells (Fig. 4a, b). Furthermore, whether TUG1 and miR-216b-5p modulation affected epithelial-to-mesenchymal transition (EMT) in HCC cells was evaluated. As displayed in Fig. 4c, d, silencing TUG1 up-regulated E-cadherin expression, and down-regulated the expressions of N-cadherin and Vimentin. Notably, these si-TUG1 effects could be weakened by co-expression of ant-miR-216b-5p. These results suggested that TUG1 regulated cell metastasis in part mediated by miR-216b-5p.

### DLX2 directly interacted with miR-216b-5p

**Fig. 5:**
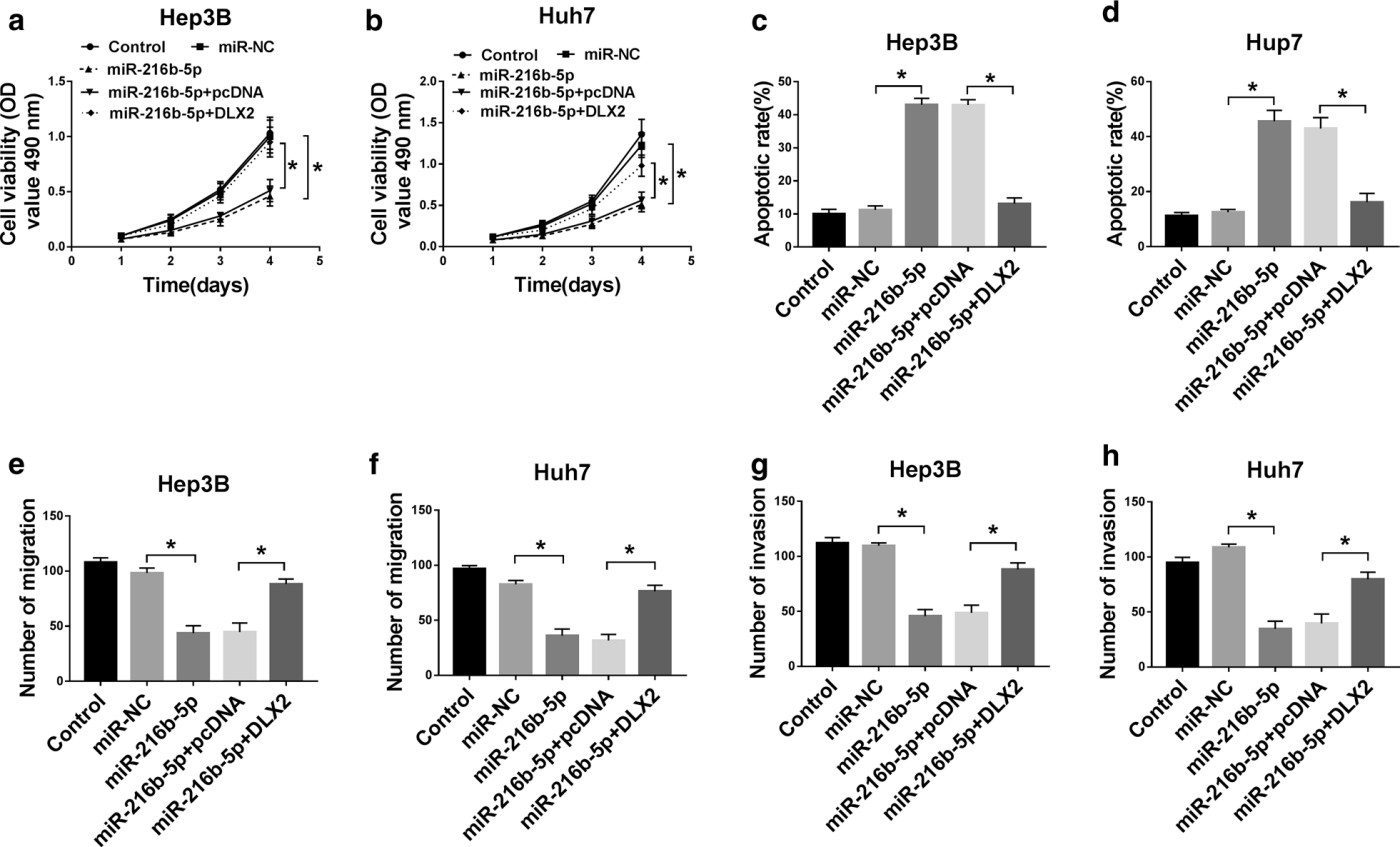
DLX2 was a direct target of miR-216b-5p in HCC. **a** The putative binding sites of DLX2 and miR-216b-5p were predicted by TargetScan. **b**, **c** The luciferase activity of Hep3B and Huh7 cells co-transfected DLX2-WT or DLX2-MUT with miR-216b-5p or miR-NC was detected. **d**, **e** The mRNA and protein expression levels of DLX2 in Hep3B and Huh7 cells transfected with miR-216b-5p or miR-NC were detected. **f**, **g** The mRNA and protein expression levels of DLX2 in Hep3B and Huh7 cells transfected with anti-miR-216b-5p or anti-miR-NC were detected (**h**–**k**). The mRNA and protein expression levels of DLX2 were measured in HCC tissues and cells. **l** Relationship between DLX2 and miR-216b-5p expression in HCC tissues was analyzed (R^2^ = 0.6632, *P *< 0.0001). *P < 0.05

Distal-less homeobox 2 (DLX2) has been reported was overexpressed in HCC and was associated with poor prognosis [23]. Meanwhile, it is predicted by TargetScan that there were binding sites between miR-216b-5p and DLX2 3′ UTR (Fig. 5a). Luciferase activity assay showed that miR-216b-5p could markedly reduce the fluorescence intensity of the DLX2-WT, but it could not notably reduce that of the DLX2-MUT in Hep3B and Huh7 cells (Fig. 5b, c). What’s importantly, qRT-PCR and Western blot analysis in Hep3B and Huh7 cells showed that the mRNA and protein expression of DLX2 were sharply reduced by the miR-216b-5p transfection and steeply elevated by the anti-miR-216b-5p transfection (Fig. 5d–g). Additionally, compared with normal tissues and cell line THLE-2, the mRNA and protein expression levels of DLX2 were enormously enhanced in HCC tissues and cell lines Hep3B and Huh7 (Fig. 5h–k). A noteworthy inverse interplay between DLX2 and miR-216b-5p was also proved (Fig. 5l). Our results showed that miR-216b-5p could negatively regulate DLX2 in HCC.

### Exogenous expression of DLX2 reversed the effects of miR-216b-5p on HCC cells

**Fig. 6:**
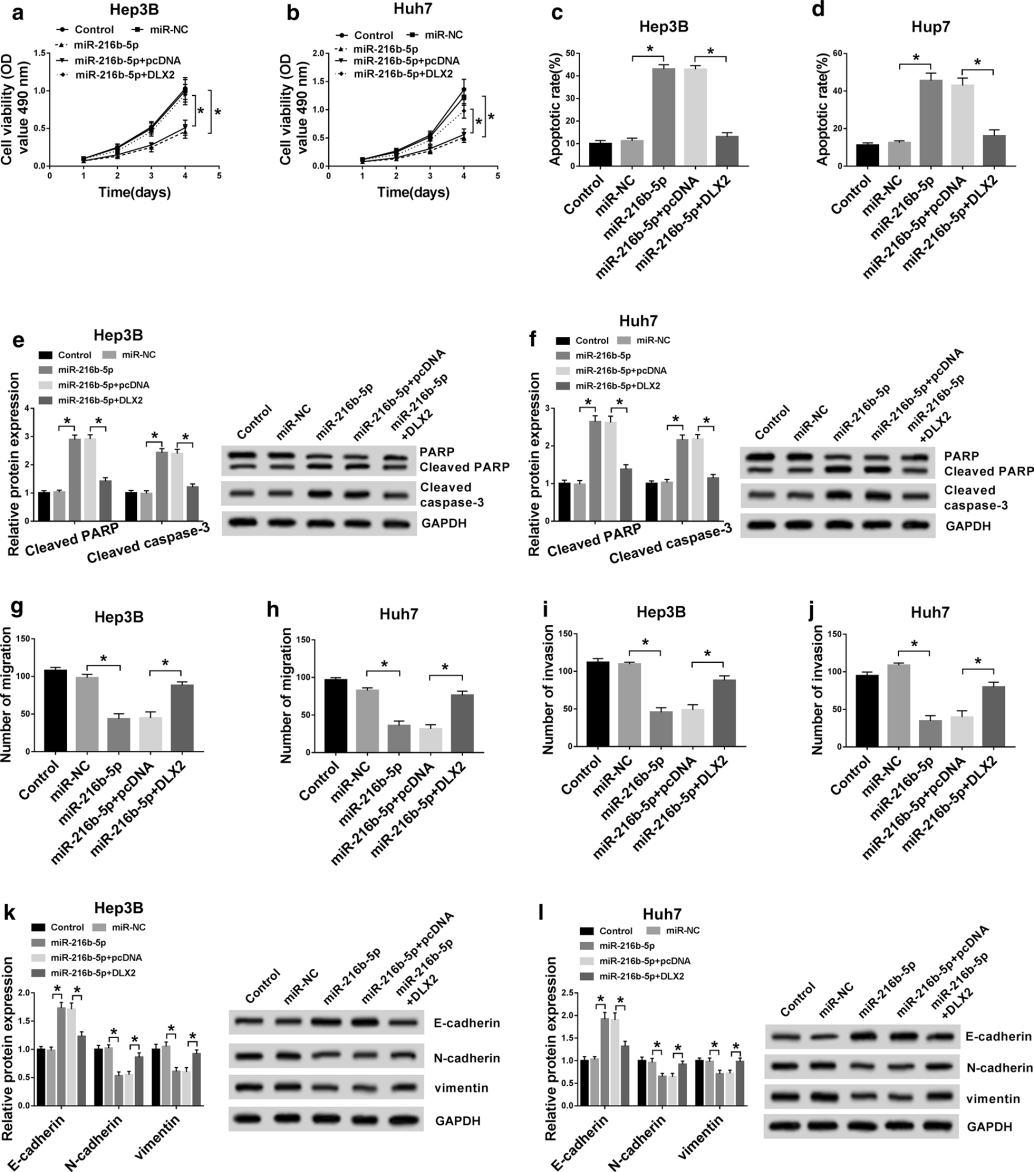
Overexpression of DLX2 could reverse the miR-216b-5p effects on proliferation, apoptosis, migration and invasion in Hep3B and Huh7 cells. Hep3B and Huh7 cells were transfected with control, miR-NC, miR-216b-5p, miR-216b-5p + pcDNA or miR-216b-5p + DLX2. **a**–**d** The cell proliferation and apoptosis were detected by MTT and Flow cytometry. **e**, **f** The protein levels of PARP, cleaved PARP and cleaved caspase-3 were measured by western blot. **g**–**j** The migration and invasion of transfected Hep3B and Huh7 cells were detected through Transwell assay. **k**, **l** The protein levels of E-cadherin, N-cadherin and Vimentin were assesses by western blot. *P < 0.05

To investigate whether miR-216b-5p regulated the progression of HCC cells by targeting DLX2, the cell function recovery experiments were detected by MTT, Annexin V-FITC/PI and transwell assays. The results showed that overexpression of miR-216b-5p in Hep3B and Huh7 cells could repress cell proliferation (Fig. 6a, b) and elevate apoptosis (Fig. 6c, d), and these effects of miR-216b-5p could be restored by overexpression of DLX2. Furthermore, the promotion effects of miR-216b-5p on levels of cleaved PARP and cleaved caspase-3 were abolished by overexpressing DLX2 (Fig. 6e, f). Similarly, overexpression of DXL2 restored the inhibitory effects of miR-216b-5p on migration and invasion of Hep3B and Huh7 cells (Fig. 6g–j), and the effects of miR-216b-5p on protein levels of E-cadherin, N-cadherin and Vimentin were counteracted when DXL2 was overexpressed (Fig. 6k, l). These date suggested that miR-216b-5p was able to regulate the growth and metastasis of HCC cells by targeting DLX2.

### TUG1 up-regulated the expression of DLX2 by sponging miR-216b-5p in HCC cells

**Fig. 7:**
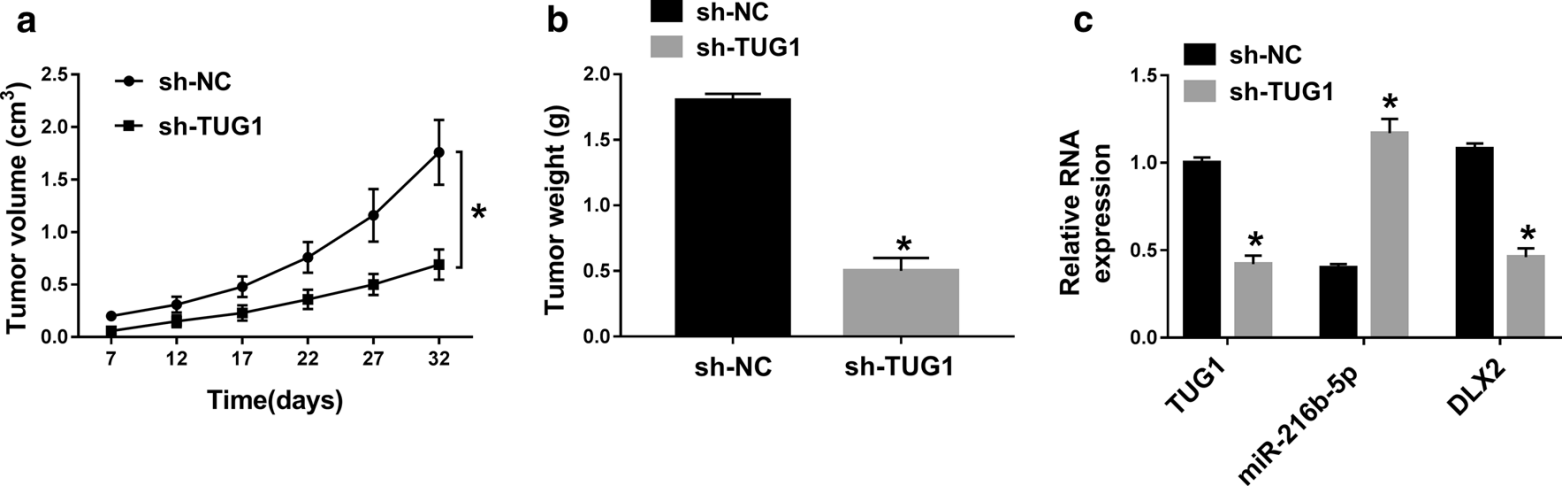
TUG1 could regulate the expression of DLX2 by sponging miR-216b-5p in HCC cells. **a**–**d** The mRNA and protein expression levels of DLX2 in Hep3B and Huh7 cells were measured by RT-qPCR and western blot transfected with control, NC, TUG1, TUG1 + miR-NC or TUG1 + miR-216b-5p. *P < 0.05

Next, we continue to explore whether TUG1 could regulate the expression of DLX2 by competitively sponging miR-216b-5p, qRT-PCR and western blot were performed. The results showed that overexpression of TUG1 of Hep3B and Huh7 cells up-regulated the mRNA and protein expression levels of DLX2, and this promotion could be regained by adding miR-216b-5p mimics (Fig. 7a–d). The results indicated that TUG1 could promote the expression of DLX2 by competitively sponging miR-216b-5p in HCC cells.

### TUG1′s oncogenic activity was in part through the regulation of miRNA-216b-5p/DLX2 in vivo

**Fig. 8:**
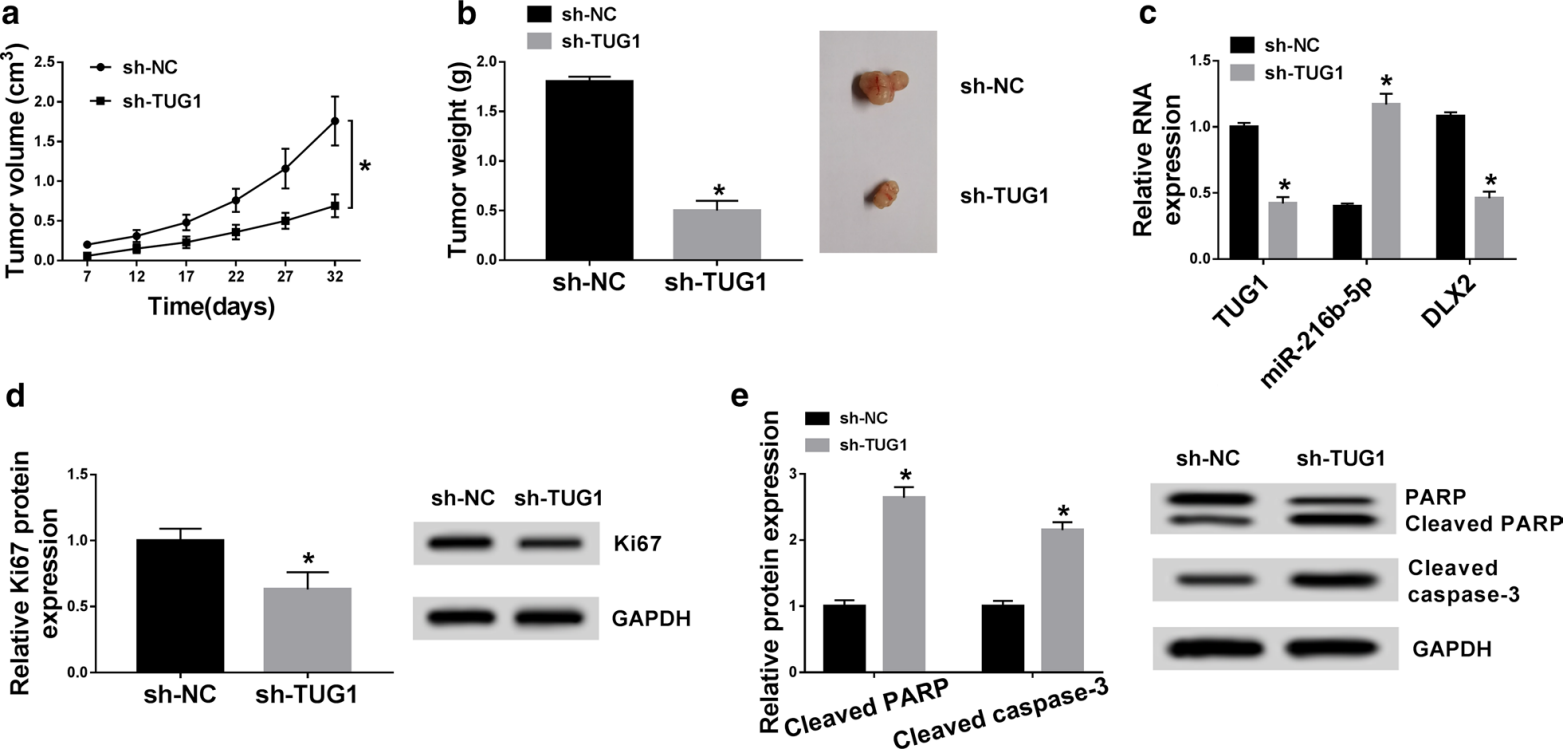
Knockdown of TUG1 could inhibit tumor growth of HCC in vivo. **a** Tumor volume was measured after injection of Huh7 cells transfected with sh-TUG1 or sh-NC. **b** Tumor weight was measured. **c** The levels of TUG1, miR-216b-5p and DLX2 in tumor tissues were detected by qRT-PCR. **d**, **e** The protein levels of Ki67, Cleaved PARP and Cleaved caspase-3 in tumor tissues were determined by western blot *P < 0.05

To further examine the oncogenic activity of TUG1, we stably transfected Huh7 cells (4 × 10^6^ cells per injection site) with sh-TUG1 or sh-NC. The cells were then injected subcutaneously into nude mice. A week later, the tumor volume was measured every 5 days and mice were euthanized after five consecutive measurements. We observed a decrease in tumor growth in the sh-TUG1 group compared with the sh-NC group by the size and weight of the tumor (Fig. 8a, b). Furthermore, the expression of TUG1, miR-216b-5p and DLX2 in resected tumor tissues was measured, compared to the sh-NC group, TUG1 and DLX2 were decreased and miR-216b-5p was increased in the sh-TUG1 group (Fig. 8c). Besides, western blot results showed that the expression of proliferation marker Ki67 was decreased in tumors of sh-TUG1 group, while the expressions of apoptotic proteins cleaved PARP and cleaved caspase-3 were increased (Fig. 7d, e). Above results indicated that TUG1 could promote tumor growth by miRNA-216b-5p/DLX2 in vivo.

## Discussion

HCC is a highly vascularized tumor with increased mortality due to its rapid metastasis and less targeted drugs [24]. Hence, exploring the pathogenesis of HCC and the potential molecular regulation mechanism could not be slacked. Thousands of lnRNAs have been found to be associated with cancers, some of which play a crucial part in the development and progression of HCC [25]. As a new lncRNA that has just been discovered, TUG1 has been recognized as an oncogene in most cancers to involve in the regulation of cancer progression [26, 27]. However, there are few studies on the molecular regulation mechanism of TUG1 on HCC.

Our results validated that TUG1 was drastically augmented in HCC tissues and cell lines. Next, we discovered that TUG1 knockdown retarded cell growth and metastasis, and induced apoptosis in HCC cells in vitro. Furthermore, silencing TUG1 blocked the growth of HCC tumors in vivo. To sum up, these date manifested a tumorigenic gene role of TUG1 in HCC. A previous study reported that knockdown of TUG1 markedly retarded cell growth, metastasis and glycolysis in HCC [28]. This implied that TUG1 might regulate other behaviors of HCC cells, which requires further study.

Most studies have made clear that lncRNAs were taken part in cancer progression by regulating the function of miRNAs. He et al. [29] found that TUG1 could promote HCC progression and ZEB1 expression by sponging miR-142-3p. More than that, the research has shown that mutual effect between TUG1 and miR-144 was given rise to proliferation and migration of HCC cells through activating the JAK2/STAT3 pathway [30]. Here, we validated that miR-216b-5p was notably declined in HCC tissues and cell lines. Moreover, TUG1 could negatively regulate miR-216b-5p expression in Hep3B and Huh7 cells. An opposite interplay between TUG1 and miR-216b-5p expression was also proved. The luciferase reporter assay also demonstrated that TUG1 could directly bind to miR-216b-5p through a complementary sequence in HCC cells. Many studies have reported that miR-216b-5p was a tumor suppressor with low expression in many cancers [31]. For example, miR-216b-5p as a tumor-inhibiting factor to hinder proliferation by targeting TPT1 in pancreatic cancer cells [32]. A recent study also reported that LINC00152 could modulate the proliferation of cervical cancer (CC) cells through elevating HOXA1 expression level via sponging miR-216b-5p [33]. Taken these into account, we believed that TUG1 played a carcinogenic role in HCC through retarding miR-216b-5p expression.

The distal-free homeobox (DLX) gene family has been supported to be involved in a lot of physiological and pathological events [34]. Among them, DLX2 plays a crucial role in the development of many human cancers, including glioma and gastric adenocarcinoma cancers [35, 36]. A previous study showed that DLX2 promoted the proliferation of HCC cells [23]. In our study, DLX2 was considered as a target of miR-216b-5p in HCC cells. Notably, miR-216b-5p positively regulated DLX2 abundance in HCC cells and a negative correlation between them was also observed in HCC tissues. It’s worth mentioning that DLX2 could recover the suppressive impacts of miR-216b-5p on the proliferation, migration, invasion and the stimulative impact on the apoptosis of HCC cells. What’s importantly, TUG1 up-regulated the expression of DLX2 by competitively sponging miR-216b-5p in vitro and in vivo.

## Conclusion

**Fig. 9:**
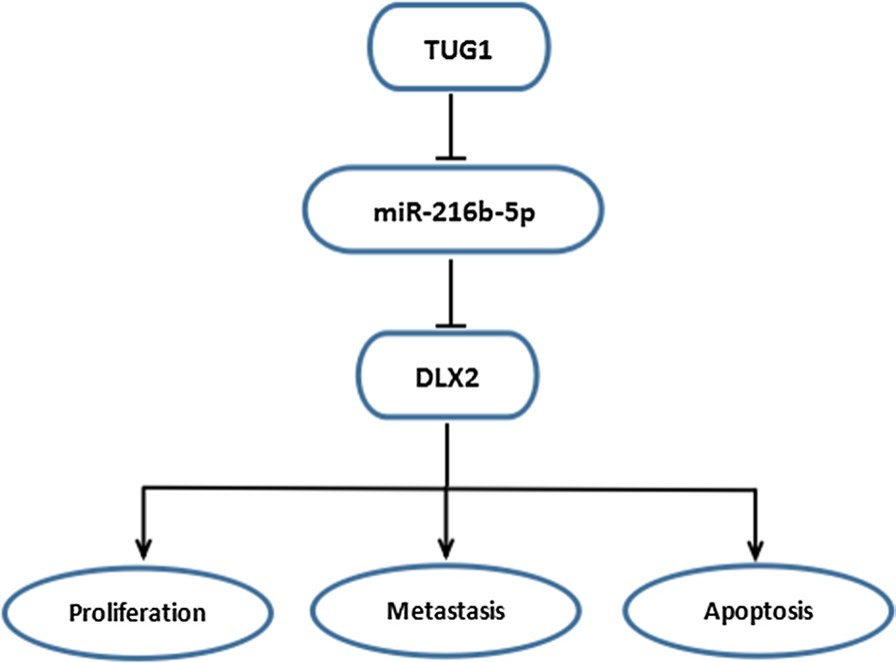
The schematic diagram of TUG1 in HCC cells

In conclusion, our research pointed to that TUG1 played a crucial part in HCC progression through regulation of DLX2 by sponging miR-216b-5p. Importantly, TUG1 was found to be regulated cell proliferation, apoptosis and metastasis via regulating miR-216b-5p/DLX2 axis (Fig. 9). Targeting the TUG1-miR-216b-5p-DLX2 network might be a new direction for the treatment of HCC.

## Data Availability

All data generated or analysed during this study are included in this published article [and its additional files].

## References

[1] Yang JD, Roberts LR (2010). Hepatocellular carcinoma: a global view. Nat Rev Gastroenterol Hepatol..

[2] Hefaiedh R, Sabbegh M, Ennaifer R, Romdhane H, Ben HN, Belhadj N, Gharbi L, Khalfallah T (2014). Percutaneous treatment versus hepatic resection for the treatment of small hepatocellular carcinoma. Tunis Med.

[3] Bosch FX, Ribes J, Díaz M, Cléries R (2004). Primary liver cancer: worldwide incidence and trends. Gastroenterology.

[4] Zhou L, Wen J, Huang Z, Nice EC, Huang C, Zhang H, Li Q (2017). Redox proteomics screening cellular factors associated with oxidative stress in hepatocarcinogenesis. Proteomics Clin Appl..

[5] Khoury S, Tran N (2015). Circulating microRNAs: potential biomarkers for common malignancies. Biomark Med.

[6] Cui M, You L, Ren X, Zhao W, Liao Q, Zhao Y (2016). Long non-coding RNA PVT1 and cancer. Biochem Biophys Res Commun.

[7] Iyer MK, Niknafs YS, Malik R, Singhal U, Sahu A, Hosono Y, Barrette TR, Prensner JR, Evans JR, Zhao S (2015). The landscape of long noncoding RNAs in the human transcriptome. Nat Genet.

[8] Hibner G, Kimsa-Furdzik M, Francuz T (2018). Relevance of microRNAs as potential diagnostic and prognostic markers in colorectal cancer. Int J Mol Sci.

[9] Muhammad N, Bhattacharya S, Steele R, Ray RB (2016). Anti-miR-203 suppresses ER-positive breast cancer growth and stemness by targeting SOCS3. Oncotarget..

[10] Ergun S, Oztuzcu S (2015). Oncocers: ceRNA-mediated cross-talk by sponging miRNAs in oncogenic pathways. Tumor Biol.

[11] Xu T, Liu C, Li T, Zhang Y, Zhao Y (2019). LncRNA TUG1 aggravates the progression of prostate cancer and predicts the poor prognosis. Eur Rev Med Pharmacol Sci.

[12] Li T, Liu Y, Xiao H, Xu G (2017). Long non-coding RNA TUG1 promotes cell proliferation and metastasis in human breast cancer. Breast Cancer..

[13] Liu L, Chen X, Zhang Y, Hu Y, Shen X, Zhu W (2017). Long non-coding RNA TUG1 promotes endometrial cancer development via inhibiting miR-299 and miR-34a-5p. Oncotarget..

[14] Zhu J, Shi H, Liu H, Wang X, Li F (2017). Long non-coding RNA TUG1 promotes cervical cancer progression by regulating the miR-138-5p-SIRT1 axis. Oncotarget..

[15] Huang M-D, Chen W-M, Qi F-Z, Sun M, Xu T-P, Ma P, Shu Y-Q (2015). Long non-coding RNA TUG1 is up-regulated in hepatocellular carcinoma and promotes cell growth and apoptosis by epigenetically silencing of KLF2. Mol Cancer.

[16] Sanchez-Mejias A, Tay Y (2015). Competing endogenous RNA networks: tying the essential knots for cancer biology and therapeutics. J Hematol Oncol.

[17] He J, Sun M, Geng H, Tian S (2019). Long non-coding RNA Linc00518 promotes paclitaxel resistance of the human prostate cancer by sequestering miR-216b-5p. Biol Cell.

[18] Fang T, Fang Y, Xu X, He M, Zhao Z, Huang P, Yuan F, Guo M, Yang B, Xia J (2019). *Actinidia chinensis* Planch root extract attenuates proliferation and metastasis of hepatocellular carcinoma by inhibiting epithelial-mesenchymal transition. J Ethnopharmacol.

[19] Pan D, Jia Z, Li W, Dou Z (2019). The targeting of MTDH by miR1455p or miR1453p is associated with prognosis and regulates the growth and metastasis of prostate cancer cells. Int J Oncol.

[20] Huang Z, Li Q, Luo K, Zhang Q, Geng J, Zhou X, Xu Y, Qian M, Zhang JA, Ji L (2019). miR-340-FHL2 axis inhibits cell growth and metastasis in ovarian cancer. Cell Death Dis..

[21] Zhang J, Xu S, Xu J, Li Y, Zhang J, Zhang J, Lu X (2019). miR-767-5p inhibits glioma proliferation and metastasis by targeting SUZ12. Oncol Rep.

[22] Yi C, Wan X, Zhang Y, Fu F, Zhao C, Qin R, Wu H, Li Y, Huang Y (2018). SNORA42 enhances prostate cancer cell viability, migration and EMT and is correlated with prostate cancer poor prognosis. Int J Biochem Cell Biol.

[23] Liu J, Cui X, Qu L, Hua L, Wu M, Shen Z, Lu C, Ni R (2016). Overexpression of DLX2 is associated with poor prognosis and sorafenib resistance in hepatocellular carcinoma. Exp Mol Pathol.

[24] Li D, Kang J, Golas BJ, Yeung VW, Madoff DC (2014). Minimally invasive local therapies for liver cancer. Cancer Biol Med.

[25] Klingenberg M, Matsuda A, Diederichs S, Patel T (2017). Non-coding RNA in hepatocellular carcinoma: mechanisms, biomarkers and therapeutic targets. J Hepatol.

[26] Li Y, Zheng D, Pan L, Dai Y, Cai S, Zhao L, Zhu H (2019). Knockdown of TUG1 by shRNA inhibited renal cell carcinoma formation by miR-299–3p/VEGF axis in vitro and in vivo. Eur J Pharmacol.

[27] Li B, Shen S, Zhang W, Qi T, Hu Q, Cheng Y (2018). Long non-coding RNA TUG1 as a potential novel biomarker for predicting the clinical outcome of cancer patients: a meta-analysis. Clin Lab.

[28] Lin YH, Wu MH, Huang YH, Yeh CT, Cheng ML, Chi HC, Tsai CY, Chung IH, Chen CY, Lin KH (2018). Taurine up-regulated gene 1 functions as a master regulator to coordinate glycolysis and metastasis in hepatocellular carcinoma. Hepatology.

[29] He C, Liu Z, Jin L, Zhang F, Peng X, Xiao Y, Wang X, Lyu Q, Cai X (2018). lncRNA TUG1-Mediated Mir-142-3p downregulation contributes to metastasis and the epithelial-to-mesenchymal transition of hepatocellular carcinoma by targeting ZEB1. Cell Physiol Biochem.

[30] Lv J, Kong Y, Gao Z, Liu Y, Zhu P, Yu Z (2018). LncRNA TUG1 interacting with miR-144 contributes to proliferation, migration and tumorigenesis through activating the JAK2/STAT3 pathway in hepatocellular carcinoma. Int J Biochem Cell Biol.

[31] Sun S, Li W, Ma X, Luan H (2019). Long noncoding RNA LINC00265 promotes glycolysis and lactate production of colorectal cancer through regulating of miR-216b-5p/TRIM44 Axis. Digestion..

[32] You Y, Tan J, Gong Y, Dai H, Chen H, Xu X, Yang A, Zhang Y, Bie P (2017). MicroRNA-216b-5p functions as a tumor-suppressive RNA by targeting TPT1 in pancreatic cancer cells. J Cancer..

[33] Zheng J, Du X, Wang H, Zhou L, Wang Y, Zhang L, Xu H, Zhang J, Hu Z (2019). Long non-coding RNA 00152 promotes cell proliferation in cervical cancer via regulating miR-216b-5p/HOXA1 axis. Eur Rev Med Pharmacol Sci.

[34] Suh Y, Obernier K, Hölzl-Wenig G, Mandl C, Herrmann A, Wörner K, Eckstein V, Ciccolini F (2009). Interaction between DLX2 and EGFR regulates proliferation and neurogenesis of SVZ precursors. Mol Cell Neurosci.

[35] Yan Z-H, Bao Z-S, Yan W, Liu Y-W, Zhang C-B, Wang H-J, Feng Y, Wang Y-Z, Zhang W, You G (2013). Upregulation of DLX2 confers a poor prognosis in glioblastoma patients by inducing a proliferative phenotype. Curr Mol Med.

[36] Tang P, Huang H, Chang J, Zhao G-F, Lu M-L, Wang Y (2013). Increased expression of DLX2 correlates with advanced stage of gastric adenocarcinoma. World J Gastroenterol.

